# A Unique Cytoplasmic–Nuclear Interaction in Sunflower (*Helianthus annuus* L.) Causing Reduced-Vigor Plants and the Genetics of Vigor Restoration

**DOI:** 10.3389/fpls.2020.01010

**Published:** 2020-07-10

**Authors:** Zhao Liu, Wei Gu, Gerald J. Seiler, Chao-Chien Jan

**Affiliations:** ^1^ Department of Plant Sciences, North Dakota State University, Fargo, ND, United States; ^2^ Institute of Plant Protection, Postdoctoral Program, Heilongjiang Academy of Agricultural Sciences, Harbin, China; ^3^ USDA-ARS, Edward T. Schafer Agricultural Research Center, Fargo, ND, United States

**Keywords:** *Helianthus*, vigor-reducing cytoplasms, vigor restoration genes, SNP, gene mapping

## Abstract

Wild *Helianthus* species are an important genetic resource for sunflower improvement, but sometimes there are adverse interactions between the wild and cultivated sunflowers. This study reports the inheritance of reduced vigor and its restoration resulting from an interaction of perennial *Helianthus* cytoplasms with nuclear genes of cultivated sunflower lines. The large number of vigor restoration (*V*) genes identified in cultivated lines are all located at the same locus, designated *V_1_*, suggesting a common origin of these genes. Additional *V* genes derived from the wild perennial species *H. giganteus* L. and *H. hirsutus* Raf. are located at a different locus than *V_1_*, designated *V_2_*. A major difference between the wild annual *Helianthus* cytoplasms and perennial cytoplasms is the lack of the vigor-reducing cytoplasms, but surprisingly *V* genes were observed in wild annual *H. annuus* L. and *H. petiolaris* Nutt. which were at the same locus as *V_1_*. A common vigor-reducing cytoplasmic effect of the perennial *Helianthus* species and the existence of a common vigor restoration *V* gene in most perennial *Helianthus* species could be explained as a result of vigor selection during *Helianthus* speciation. *V_1_* was mapped on linkage group (LG) 7 of the sunflower genome, using an F_2_ population derived from MOL-RV/HA 821. *V_1_* co-segregated with an InDel marker ZVG31, with three single-nucleotide polymorphism (SNP) markers, SFW01024, SFW07230, and SFW00604, located above it on the map at a genetic distance of 0.8 cM, and another SNP marker, SFW08671, below it at a distance of 0.4 cM. The physical distance between the two closest flanking SNP markers corresponds to 0.56 and 1.37 Mb on the HA 412-HO and XRQ assemblies, respectively. The tightly linked markers will help select normal vigor progenies when using perennial *Helianthus* cytoplasms in a breeding program, which will also provide a basis for studying the mechanism of the cytonuclear interaction, and the speciation of annual and perennial *Helianthus* species.

## Introduction

Crop wild relatives (CWR) are an important genetic resource for crop improvement to biotic and abiotic stresses in many crops, such as wheat, rice, maize, barley, oat, cotton, and soybean (Yumurtaci, 2015; Mammadov et al., 2018). Cultivated sunflower (*Helianthus annuus* L., 2n = 2× = 34) is one of the few crops native to the United States. The *Helianthus* genus is well known for its taxonomic complexity, which includes 53 species (14 annual and 39 perennial) and 19 subspecies (Seiler et al., 2017; Anderson et al., 2019). The annual species are all diploid (including cultivated sunflower), and the perennial species include 26 diploid, three tetraploid, seven hexaploid, and three mixaploid species. As with other CWR, wild *Helianthus* species represent a large unexploited gene pool with genetic variation for different traits, such as resistance to Sclerotinia, Phomopsis, rust, and downy mildew diseases, and parasitic broomrape (Seiler et al., 2017). They are also a source of new cytoplasmic male sterility (CMS) for sunflower improvement. All the annual *Helianthus* species, except *Helianthus agrestis* Pollard, can be hybridized with cultivated sunflower using classical crossing methods (Seiler et al., 2017). However, utilization of the perennial diploid species which represent half of the *Helianthus* genus, is limited by poor crossability and F_1_ sterility in wild × cultivated interspecific hybrids. Development of a two-stage embryo rescue technique and a colchicine treatment of seedlings to double the chromosome number have minimized these problems and made it possible to produce interspecific amphiploids (Amp) (Jan, 1988; Sukno et al., 1999). These amphiploids have proven to be extremely valuable in transferring resistance to *Orobanche cumana* Wallr. (broomrape) race F, and in the introgression of fertility restoration genes into cultivated sunflower (Jan and Fernandez-Martinez, 2002; Jan et al., 2002; Feng and Jan, 2008; Liu et al., 2013).

Another limitation in the use of the perennial wild species is the existence of adverse cytonuclear interactions. Previously, reduced-vigor (RV) plants were observed in backcross progenies of an inbred line HA 89 in the cytoplasms of five perennial *Helianthus* species (*H*. *mollis* Lam., *H. maximiliani* Schrad., *H. grosseserratus* Mar., *H*. *divaricatus* L., and *H*. *angustifolius* L.) (Jan, 1992). The characteristics of RV plants included pale-green leaves, significantly reduced plant height, head diameter, seed weight, percent seed set, net photosynthesis, total leaf chlorophyll, and delayed flowering. The plant vigor reduction effects varied among the different cytoplasms. A cytoplasmic component of these effects has been confirmed by the occurrence of all-normal progenies in crosses of HA 89 with pollen from RV plants. Genetic studies suggested that each of the five species has a single dominant nuclear gene controlling plant vigor restoration (Jan, 1992). In addition, the segregation ratios of normal (N) to RV plants observed in the F_2_ progeny of diallel crosses among normal plants with heterozygous or homozygous vigor restoration genes derived from the above five interspecific crosses indicated a common perennial cytoplasmic deficiency in these wild perennial species, and that a common vigor restoration gene could restore the plant vigor (Jan, 1995).

The cytoplasmic genome of plants contains 120–140 genes in the mitochondria and 95–100 genes in the chloroplast. Both chloroplasts and mitochondria require the import of nuclear-encoded proteins for organelle biogenesis (Levin, 2003). The results of above reciprocal crosses indicated that the phenotypes of RV plants in the progenies of the five perennial *Helianthus* species (Jan, 1992) may arise from cytonuclear interactions between the nuclear genome of the annual *H. annuus* and the cytoplasms of the perennials. Cytonuclear incompatibilities may play a role in establishing reproductive isolation among these species (Burton et al., 2013). The study of the reciprocal F_1_ hybrids and backcross families of *H. annuus* and *H. petiolaris* in xeric and mesic habitats of the parental species suggested that the parental species’ cytoplasms were strongly locally adapted and that cytonuclear interactions significantly affected the fitness and architecture of hybrid plants (Sambatti et al., 2008). Using a target enrichment approach, Stephens et al. (2015) studied phylogeny relationships across 37 diploid *Helianthus* species/subspecies with a total of 103 accessions using 170 nuclear genes and the chloroplast sequences. Their phylogeny analysis with nuclear genes supported three major clades including a large annual clade, a southeastern perennial clade, and another clade of primarily large-statured perennials. A rapid radiation and/or high levels of reticulate evolution among perennial *Helianthus* species was suggested in their study. Later, Lee-Yaw et al. (2019) analyzed the phylogenetic relationships among annual *Helianthus* species and individuals, using nuclear SNPs and chloroplast genomes sequences. The two perennial species *H. nuttallii* Torr. & A. Gray and *H. maximiliani* and one more distantly related genus *Phoebanthus grandiflora* Torr. & A. Gray were separated in a distinct clade when used as outgroups for the annual *Helianthus* species. These phylogenetic analyses indicated the clear distinction between the nuclear and chloroplast of annual and perennial *Helianthus* species (Stephens et al., 2015; Lee-Yaw et al., 2019).

Recently, RV and normal progenies have been observed in nine additional perennial *Helianthus* species when the wild species were used as maternal parents crossed with the cultivated sunflower inbred lines HA 89 or HA 410, with the goal of transferring Sclerotinia resistance and other useful genes from wild perennial *Helianthus* species into cultivated sunflower, including *H*. *giganteus* L., *H*. *hirsutus* Raf., *H*. *salicifolius* A. Dietr., *H*. *pauciflorus* Nutt., *H*. *californicus* DC., *H*. *nuttallii* T.& G., *H*. *occidentalis* Riddell subsp*. plantagineus* (T. & A. Gray) Heiser, *H*. *schweinitzii* T. & G., and *H*. *tuberosus* L. Therefore, reduced plant vigor and its restoration are commonly observed in utilizing CWR for sunflower improvement. The objectives of this study were to: 1) further examine reduced plant vigor and vigor restoration (*V*) genes for the vigor-reducing cytoplasms of wild perennial species with respect to cultivated sunflower, wild annual *Helianthus* species, and perennial *H. giganteus* and *H. hirsutus*; 2) examine the relationships among the *V* genes in cultivated sunflower, wild perennial and annual *Helianthus* species, and determine the inheritance of vigor restoration; and 3) map the common *V* gene from cultivated sunflower to a genetic map using an F_2_ population derived from the cross of MOL-RV/HA 821.

## Materials and Methods

### Plant Materials

Eight wild perennials plus two wild annual *Helianthus* species were used in this study. The wild perennials included *H. mollis*, *H. giganteus*, *H. maximiliani*, *H. grosseserratus*, *H. angustifolius*, *H*. *salicifolius*, *H. hirsutus*, and *H. pauciflorus*. The wild annuals included *H. annuus* and *H. petiolaris*. One alloplasmic line with *H*. *mollis* cytoplasm (MOL-RV), 14 cultivated sunflower lines (HA 89, HA 821, HA 234, RHA 271, RHA 296, RHA 801, P21, Peredovik, VNIIMK 6540, Smena, Seneca, Issanka, Armavir 3497, and Hopi Dye), and one RV CMS line derived from interspecific cross (CMS RIGX-RV) was used to study the inheritance of the *V* genes (**Table 1**). The normal and RV progeny plants derived from seven perennials (*H. mollis*, *H. giganteus*, *H. grosseserratus*, *H. angustifolius*, *H. salicifolius*, *H. hirsutus*, and *H. pauciflorus*) and two annuals (*H. annuus* and *H. petiolaris*) were used to determine the genetics and the relationships of the *V* genes. HA 89, HA 821 and HA 234 are oilseed maintainer lines, whereas RHA 271, RHA 296 and RHA 801 are oilseed restorer lines. These inbred lines were publicly released by USDA. P21 is a nuclear male-sterile line released by the USDA and the Texas Agricultural Experiment Station in 1970 (Jan and Vick, 2006). VNIIMK 6540, VNIIMK 8931 (for HA 89), Armavir 3497, Peredovik, and Smena were varieties developed by former Soviet Union. Issanka was developed in France. Seneca and Hopi Dye are Native American Indian Landraces. CMS RIGX-RV has perennial cytoplasm from *H. pauciflorus*.

**Table 1:** List of sunflower materials used in the study.

Line	PI No.	*V* gene	Cytoplasm	Pedigree	Phenotype	Year released	Reference	
HA 89	599773	*v_1_*	*H. annuus*	VNIIMK 8931 Selection	Normal	1971	NDSUFS^a^, NPGS^b^
HA 234	599778	*V_1_*	*H. annuus*	2*Smena//HA 6/HA 8	Normal	1971	NDSUFS, NPGS
RHA 271	599786	*V_1_*	*H. petiolaris*	CMS PI 343765/HA 119//HA 62-4-5/2/T66006-2-1-31-1=T70020	Normal	1973	NDSUFS, NPGS
HA 410	603991	*v_1_*	*H. annuus*	B-Line SCL Recurrent Selection	Normal	1995	NDSUFS, NPGS
HA 821	599984	*V_1_*	*H. annuus*	HA 300 Selection (HA 300 = Peredovik 301 (PI 372172))	Normal	1983	NDSUFS, NPGS
RHA 296	552931	*V_1_*	*H. petiolaris*	RHA 274 Reselection	Normal	1973	NDSUFS, NPGS
RHA 801	599768	*v_1_*	*H. petiolaris*	Derived from a Restorer Composite	Normal	1980	NDSUFS, NPGS
P21		*V_1_*	*H. annuus*	Cultivar	Normal	1970	Jan and Vick (2006)
VNIIMK 6540	265503	*V_1_*	*H. annuus*	Open-pollinated variety	Normal	2007	NPGS; Vear (2010)
Armavir 3497	372254	*V_1_*	*H. annuus*	VNIIMK 1646	Normal	1972	NPGS; Vear et al. (2011)
Issanka	650813	*V_1_*	*H. annuus*	Cultivar	Normal	2007	NPGS
Peredovik	307937	*V_1_*	*H. annuus*	Open-pollinated variety	Normal	1965	NPGS; Vear (2010)
Smena	372258	*V_1_*	*H. annuus*	Cultivar	Normal	1972	NPGS
Seneca	369360	*v_1_*	*H. annuus*	Landrace	Normal	1972	NPGS
Hopi Dye	432504	*V_1_*	*H. annuus*	Landrace	Normal	1978	NPGS
MOL-RV		*v_1_*	*H. mollis*	*H. mollis*/(8/9)*HA 89	RV	–	This study.
CMS RIGX-RV		*v_1_, v_2_*	*H. pauciflorus*	CMS RIGX/5*HA 89	CMS, RV	–	Jan and Russo (2002)
GRO-RV		*v_1_*	*H. grosseserratus*	*H. grosseserratus* PI 416793/8*HA 89	RV	–	This study.
ANG-RV		*v_1_*	*H. angustifolius*	*H. angustifolius*/8*HA 89 F_2_	RV	–	This study.
SAL-RV		*v_1_*	*H. salicifolius*	*H. salicifolius* Ames 30340/4*HA 410 F_2_	RV	–	This study.
HIR-RV		*v_2_*	*H. hirsutus*	*H. hirsutus* PI 547174/4*HA 410 F_4_	RV	–	This study.
PAU-RV		*v_1_*	*H. pauciflorus*	*H. pauciflorus*/3*HA 89 F_2_	RV	–	This study.
CMS GIG2	671967	*V_2_*	*H. giganteus*	*H. giganteus* 1934/6*HA 89	CMS, Normal	2014	Feng et al. (2015)
RF GIG2-MAX 1631	671969	*V_2_*	*H. giganteus*	CMS GIG2/(NMS HA 89/*H. maximiliani* 1631, Amp), F_4_	Normal	2014	Feng et al. (2015)
HIR		*V_2_*	*H. hirsutus*	*H. hirsutus* PI 547174/4*HA 410	Normal	–	This study.
ANN Bulk	Bulk	*V_1_*	*H. annuus*	Bulk of *H. annuus* PI 413161, PI 435378, PI 435417, PI 435424, PI 435432 and PI 435438	Normal	–	This study.
ANN PI 649856	649856	*V_1_*	*H. annuus*	*H. annuus* PI 649856	Normal	–	This study.
PET Bulk	Bulk	*V_1_*	*H. petiolaris*	Bulk of *H. petiolaris* PI 686914 and PI 592359	Normal	–	This study.

a NDSUFS: North Dakota State University Foundation Seedstocks, https://www.ag.ndsu.edu/fss/ndsu-varieties/usda-sunflower-inbred-lines.

b NPGS, USDA National Plant Germplasm System, https://npgsweb.ars-grin.gov/gringlobal/search.aspx

The pedigree of the lines used in the study follow the nomenclature system of Purdy et al. (1968). Briefly, the symbol “/” indicated the primary cross and the backcrosses are indicated by numerals at the “/” symbol and placed on the same side of the symbol as the recurrent parent. Then numerals and the recurrent parent are separated by an asterisk “*”. The numerals indicate the number of times the recurrent parent was used, example, (*H. giganteus*/6*HA89). The “//” symbol indicates a secondary cross. Amphiploids were also used in the pedigree designated as i.e. NMS HA89/*H. maximiliani* 1631, Amp.

To study the relationship among *V* genes from different sources, six homozygous vigor restoration sources including HA 821; RF GIG2-MAX 1631 (pedigree: CMS GIG2/(NMS HA89/*H. maximiliani* 1631, Amp) F_4_, Normal); HIR (Pedigree: *H*. *hirsutus* PI 547174/4*HA 410, F_4_, Normal); two *H. annuus* sources, PI 649856 and a bulk of PI 413161, PI 435378, PI 435417, PI 435424, PI 435432 and PI 435438; plus a bulk of two *H. petiolaris* (PI 686914 and PI 592359) were pollinated onto five homozygous RV lines (GRO-RV, ANG-RV, SAL-RV, HIR-RV, and PAU-RV) with vigor-reducing cytoplasms of *H. grosseserratus*, *H. angustifolius*, *H. salicifolius*, *H. hirsutus*, and *H. pauciflorus* in 2015. CMS GIG2 is a normal-vigor progeny (pedigree: *H. giganteus*/6*HA89, CMS, Normal) (Feng et al., 2015). The pedigrees of the materials used in the study are listed in **Table 1**.

### Progeny Test for Plant Vigor for Progenies Derived From MOL-RV and Cultivated Sunflower

Reduced-vigor progeny of *H. mollis*/8*HA 89 (MOL-RV) were grown in the greenhouse in 1998 and pollinated with 14 cultivated sunflower lines from diverse genetic backgrounds (**Table 1**). Vigor-restored normal (N) F_1_ plants were self-pollinated and F_2_ progeny evaluated in the greenhouse for plant vigor restoration under normal sunflower growth conditions in 1999. The F_2_ segregation ratios of N to RV plants were compared to hypothetical ratios using Chi-square analyses.

Eleven cultivated lines homogeneous or with a high frequency of vigor restoration genes (Jan and Ruso, 2000) were emasculated and pollinated with HA 89. All the F_1_s were self-pollinated to obtain F_2_ progenies. For each cross, 40 F_2_ progenies were planted in the greenhouse to observe the segregation of N and RV plants.

### Half-Diallel Analysis of Vigor Restoration (*V*) Genes in Restoration Lines

To tentatively test the hypothesis that the *V* genes in cultivated lines originated from a common source, HA 271, HA 234, VNIIMK 6540, Armavir 3497, Issanka, and HA 821 were included in a half-diallel cross. Testcrosses were made by pollinating CMS RIGX-RV plants with a HA 89 background with 15 F_1_s (Jan and Ruso, 2000). The use of CMS RIGX-RV plants as the female parent assured cross-pollination. The testcross progenies were evaluated in the greenhouse for plant vigor segregation.

### Molecular Mapping of the *V_1_* Gene

An F_2_ population including 124 individuals of G99/501-625 derived from MOL-RV/HA 821 was used to map the *V_1_* gene from HA 821. The N and RV segregation of the F_2_ progenies were examined in the greenhouse in 1999, and their genotypes was further confirmed by using F_3_ progeny grown in the greenhouse in 2013–2014, with 20–40 progeny seedlings each.

Genomic DNA was extracted according to the protocol of the Qiagen DNAeasy 96 Plant Kit (Qiagen, Valencia, CA, USA). The bulked segregant analysis (BSA) method was used for polymorphism screening (Michelmore et al., 1991). The two parents and the two bulks were used for screening. The two bulks included a homozygous normal bulk (Bulk-N) and a homozygous reduced vigor bulk (Bulk-RV), using equal quantities of DNA from 10 F_2_ plants for each bulk. The PCR amplification and genotyping for SSR markers followed Liu et al. (2012). PCR amplification was conducted following Tang et al. (2002) with minor modifications. The 15-µl PCR reaction mixture contained 1× PCR buffer, 2 mM MgCl_2_, 0.2 mM dNTPs, 0.27 µM each of the forward and reverse primers, 40 ng DNA and 1-unit *Taq* DNA polymerase (Qiagen). PCR amplifications were performed using the “touchdown” profile (Lai et al., 2005; Feng and Jan, 2008) in an MJ Research (Watertown, MA, USA) single or Bio-Rad (Hercules, CA, USA) single or dual 96-well thermal cycler. The PCR products were separated on a 6.5% denaturing polyacrylamide gel after denaturation at 95°C for 5 min, at 60 W for 2.0 h (1× TBE) after pre-run for 1.0 h or on a 6.5% non-denaturing polyacrylamide gel at 60 W for 1.0 h (0.5× TBE), on a CBP Scientific gel electrophoresis system. The gels were analyzed after being stained with GelRed nucleic acid gel stain (Biotium Inc., CA, USA) and scanned with a Typhoon 9410 variable mode imager (Molecular Dynamics Inc., CA, USA).

Bulked segregant analyses (Michelmore et al., 1991) were conducted for polymorphism screening using 550 SSR and expressed sequence tag (EST)-SSR primers on 17 LGs of sunflower, following the method of Liu et al. (2012). An additional 30 SSR/EST-SSR and InDel primers on the candidate LG 7 from 23 maps in the Sunflower CMap Database (http://sunflower.uga.edu/cgi-bin/cmap/map search) were used for polymorphism detection between the two parents. A total of 58 SSR/EST-SSR and InDel primers from LG 7 were used for polymorphism screening between parents and bulks. In addition, 30 sets of semi-thermal asymmetric reverse PCR (STARP) primers were designed according to the sequences of 30 SNP loci previously mapped on LG 7 (Bowers et al., 2012; Talukder et al., 2014; Hulke et al., 2015) following the method of Long et al. (2017). Each set of STARP primer included two universal priming element-adjustable primers (PEA-primers) and two asymmetrically modified allele-specific primers (AMAS-primers) in combination with one common reverse primer (Long et al., 2017). Polymorphism screening for the SNP markers was conducted among the two parents and Bulk-N. The PCR amplification system, program and product separation were performed as described in Liu et al. (2019). Briefly, the PCR program started with initial denaturation at 94°C for 3 min, followed by six cycles of 2-step touchdown PCR program, in which the Ta/e was decreased by 1°C per cycle starting at 94°C for 20 s and then 55°C for 2 min. PCR was continued with another 34 cycles of 2-step program at 94°C for 20 s and then 62°C for 2 min. The amplification was completed with a 2-min extension at 62°C. The PCR products for STARP markers were separated on a denaturing gel on IR2 4300/4200 DNA Analyzer (LI-COR, Lincoln, NE, USA). The polymorphic markers closely linked to the *V_1_* gene were used for genotyping the mapping population.

The deviation analyses of the vigor trait and marker loci were compared with the expected Mendelian ratios in the F_2_ generation using the Chi-square test. The MAPMAKER/Exp version 3.0b program (Whitehead Institute, Cambridge, MA, USA) (Lander et al., 1987) was used for linkage analysis of the phenotypes and molecular genotypes, with a minimum LOD score of 4.0 and a maximum recombination frequency of 0.30. The Kosambi mapping function was used (Kosambi, 1944), with the “error detection on” command. The linkage map was generated using MapChart 2.3 (Voorrips, 2002).

### Physical Location of *V_1_* on LG 7

The analysis of the physical location of *V_1_* and linked SNP and other markers on LG 7 was conducted by a BLASTn search against HA412.v1.1.bronze.20141015 on websites of the Sunflower Genome Database (https://sunflowergenome.org/) and the XRQ genome (https://www.heliagene.org/HanXRQ-SUNRISE/) on the INRA Sunflower Bioinformatics Resources (https://www.heliagene.org/) by using the sequences flanking the SNP markers and the sequences of other primers. The order of the linked markers on the genetic map of LG 7 was compared with those of Bowers et al. (2012) and Hulke et al. (2015).

### Vigor Restoration of Cultivated Sunflower for Progenies With *H. giganteus* Cytoplasm

*Helianthus giganteus* 1934 was pollinated by HA 89 and the F_1_ plants were obtained *via* embryo rescue in 1995. One F_1_ plant was male-sterile and backcrossed with HA 89. Segregation of N and RV plants was observed in BC_3_F_1._ Three normal BC_4_F_1_ plants were pollinated with HA 89 pollen and the BC_5_F_1_ plants were evaluated for the segregation of N and RV plants. To study the genes controlling vigor restoration and fertility restoration, a normal-vigor and fertile F_1_ plant derived from the cross CMS GIG2//CMS GIG2/(NMS HA 89/*H. maximiliani* 1631, Amp) was selfed. The F_2_ progenies were phenotyped for both vigor and male fertility. Additionally, normal-vigor CMS GIG2 (pedigree: *H. giganteus*/6*HA89, CMS, Normal) plants were pollinated by HA 821 and testcrossed using HA 89 pollen. Progeny segregation patterns were used to determine the allelism of the vigor restoration genes derived from *H. giganteus* and in HA 821.

### The F_1_ and Testcross Progeny Test for *V* Genes Derived From Different Sources

The F_1_s from crossing RV lines of HA 89 or HA 410 with five perennial *Helianthus* vigor-reducing cytoplasms of *H. grosseserratus* (GRO-RV), *H. angustifolius* (ANG-RV), *H. salicifolius* (SAL-RV), *H. hirsutus* (HIR-RV), and *H. pauciflorus* (PAU-RV) to six homozygous vigor restoration lines (HA 821, RF GIG2-MAX 1631, HIR, ANN PI 649856, ANN Bulk, and PET Bulk) were evaluated for segregation of plant vigor in 2016. Due to the discovery of homozygous *V* genes in both wild *H. annuus* (i.e. ANN Bulk and ANN PI 649856) and *H. petiolaris* (i.e. PET Bulk), partial half-diallel crosses among the six homozygous or heterozygous vigor restoration lines were established in the greenhouse in 2016, including three homozygous lines (HA 821, RF GIG2-MAX 1631, and HIR), and three heterozygous lines from ANN bulk, ANN PI 649856, and PET bulk. Then, six progeny plants from each cross were used to pollinate the CMS RIGX-RV with *H. pauciflorus* cytoplasm in 2016. Segregation of the N and RV F_2_s and testcross F_1_s was evaluated in the greenhouse in 2017. Progeny segregation of either 1N:1RV or no segregation suggests the same *V* gene for the two parents, and progeny segregation of either 1N:1RV or 3N:1RV suggests the two parents have different *V* genes. The data from progenies with segregation of 1N:1RV were not shown.

## Results

### Vigor Restoration of Cultivated Sunflower for Progenies With *H. mollis* Cytoplasm

**Figure 1:**
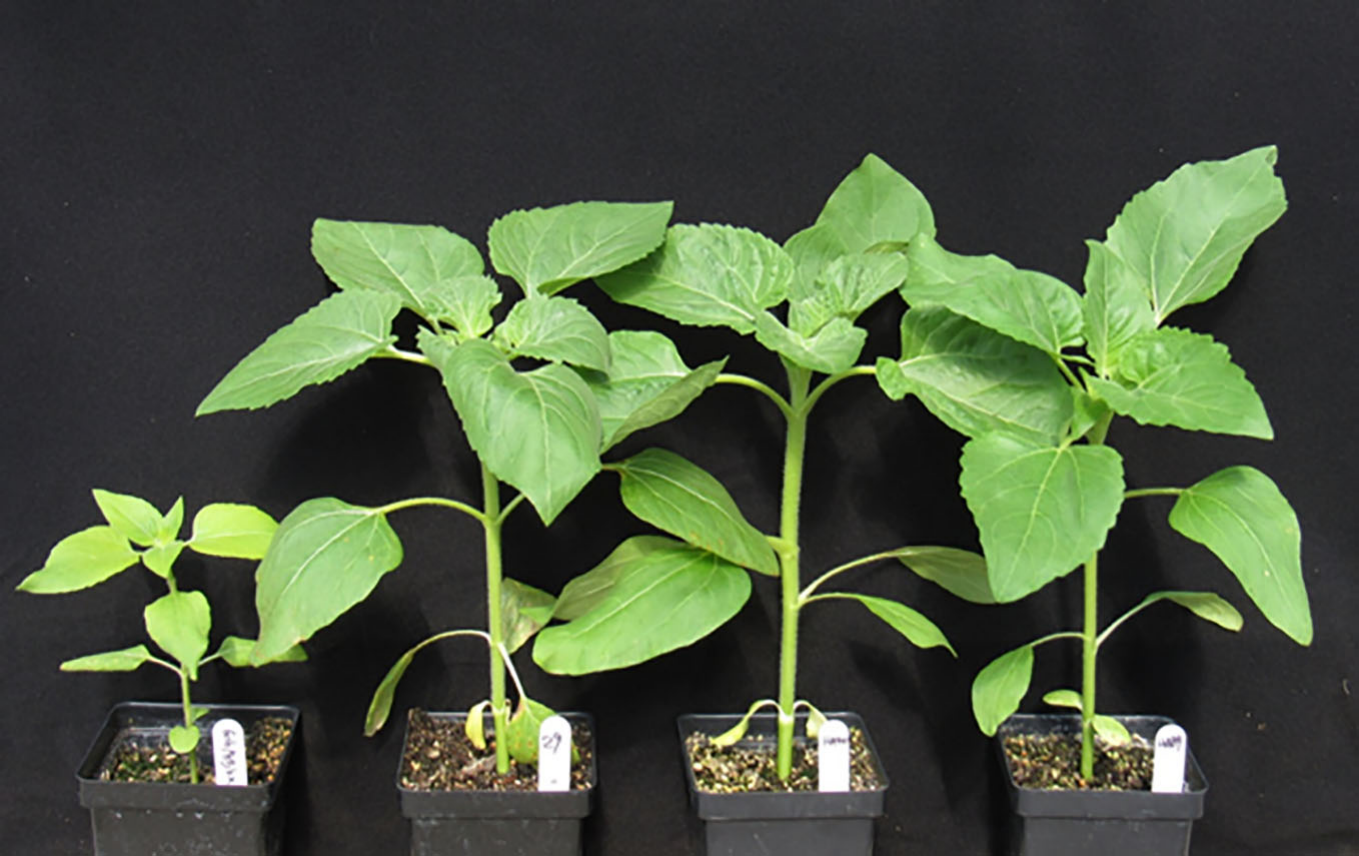
Representative reduced-vigor (RV) and normal (N) progeny plants of perennial *Helianthus* species crossed with cultivated sunflower lines at 35 days after planting. Left to right: RV progeny; normal progeny; HA 410; HA 89.

Segregation patterns of the F_1_ progenies of the RV plants with *H. mollis* cytoplasm, MOL-RV, crossed with 14 cultivated sunflower lines in 1998 are shown in **Table 2**. Typical N and RV seedlings are shown in **Figure 1**. A high frequency of vigor restoration genes was found in the crosses involving 11 cultivated sunflower lines. The crosses of MOL-RV with HA 821, HA 234, and RHA 271 produced only normal progeny, suggesting the vigor restoration (*V*) genes in those lines were homozygous. The crosses of MOL-RV with HA 89, RHA 801, and Seneca produced only RV progeny, indicating that these lines did not have dominant vigor restoration genes. In addition, another inbred line, HA 410, doesn’t contain *V* genes (data not shown). Progeny from crosses with the remaining eight lines had a high frequency of normal plants, suggesting these lines contain *V* genes, although the *V* genes may not be homozygous. Since the MOL-RV plants were emasculated over several days, this low frequency of RV progeny could also be the result of accidental self-pollination.

**Table 2:** Normal (N) and reduced-vigor (RV) plants in F_1_ progenies of MOL-RV crossed with 14 cultivated sunflower lines.

Pedigree	Number of plants	
	N	RV
MOL-RV/HA 821^†^	10	0
MOL-RV/HA 89	0	10
MOL-RV/HA 234^†^	10	0
MOL-RV/RHA 296	8	2
MOL-RV/RHA 271^†^	10	0
MOL-RV/RHA 801	0	7
MOL-RV/Peredovik	8	2
MOL-RV/Armavir 3497^†^	9	1
MOL-RV/VNIIMK 6540^†^	11	1
MOL-RV/Smena	8	1
MOL-RV/P21	10	1
MOL-RV/Issanka^†^	8	1
MOL-RV/Hopi Dye	5	5
MOL-RV/Seneca	0	11

^†^Selected lines for half-diallel crosses.

The F_2_ segregation ratios of N and RV plants of the MOL-RV line crossed with 10 cultivated sunflower lines are shown in **Table 3**. The segregation ratio of 3 N to 1 RV in nine of the 10 crosses was consistent with a single dominant gene hypothesis for control of vigor restoration. The segregation ratio of the cross MOL-RV/Armavir 3497 did not fit 3 N to 1 RV ratio (χ^2^ = 4.800, *P* = 0.028). The F_2_ plants of MOL-RV/Armavir 3497 were also tested for a segregation ratio of 15:1, which could not exclude two loci controlling the vigor restoration in Armavir 3497 (χ^2^ = 0.960, *P* = 0.327). However, the seeds used to produce reduced-vigor plants sometimes have lower germination rate compared to normal-vigor plants, so this maybe the most likely reason for the segregation ratio in this F_2_ population not fitting a 3:1 ratio. As a group, the homogeneity test with a probability of 0.241 also supports the single dominant gene hypothesis for vigor restoration.

**Table 3:** Segregation of normal (N) and reduced-vigor (RV) F_2_ plants of MOL-RV crossed with 10 cultivated sunflower lines having vigor restoration (*V*) genes.

Pedigree	Number of plants		3 N: 1 RV theoretical segregation ratio	
	N	RV	χ^2^	*P*-value
MOL-RV/HA 234	33	7	1.200	0.273
MOL-RV/RHA 271	31	9	0.133	0.715
MOL-RV/RHA 296	31	8	0.419	0.518
MOL-RV/P21	30	8	0.316	0.574
MOL-RV/Peredovik	27	13	1.200	0.273
MOL-RV/VNIIMK 6540	26	14	2.133	0.144
MOL-RV/Smena	31	9	0.133	0.715
MOL-RV/Issanka	27	11	0.316	0.574
MOL-RV/Armavir 3497*	36	4	4.800	0.028
MOL-RV/Hopi Dye	33	7	1.200	0.273
Homogeneity			11.528	0.241

*The F_2_ plants of MOL-RV/Armavir 3497 was also tested for a segregation ratio of 15:1, with χ^2^ = 0.960, P = 0.327.

### Half-Diallel Analysis of Vigor Restoration Genes in Cultivated Sunflower

The F_1_ hybrids of the 11 lines, including HA 271, HA 234, VNIIMK 6540, Armavir 3497, Issanka, HA 821, RHA 296, Peredovik, Smena, P21, and Hopi Dye, crossed with HA 89 were all N. With over 400 F_2_ progeny plants, 40 F_2_ progeny plants for each cross, there was not a single RV plant observed (data not shown). This suggested that there is no reduced vigor problem caused by cytonuclear interaction in these cultivated sunflower lines. The F_1_ progeny of the half-diallel crosses among six cultivated lines all had normal vigor, i.e., HA 271, HA 234, VNIIMK 6540, Armavir 3497, Issanka, and HA 821. The testcross progenies of the half-diallel crossed F_1_s among HA 271, HA 234, VNIIMK 6540, Armavir 3497, Issanka, and HA 821 onto the RV CMS RIGX were all normal, except for the testcross with VNIIMK 6540/Armavir 3497 F_1_, where a segregation ratio of 1 N to 1 RV plant was observed (Jan and Ruso, 2002). The 1 N to 1 RV segregation in the testcross using VNIIMK 6540/Armavir 3497 pollen could be due to a heterozygous F_1_ plant that resulted from a rare heterozygous parent. Therefore, the progeny test results suggested that all these lines possess the same *V* gene, designated *V_1_*.

### Molecular Mapping of *V_1_*Gene on LG 7

The F_2_ population G99/501-625 derived from MOL-RV/HA 821 was used to map the *V_1_* gene from HA 821. The 124 individuals in this population included 28 homozygous N, 59 heterozygous N, and 33 RV plants, confirmed by the progeny test, with four individuals not having enough seeds for progeny testing. Chi-square analysis indicated that the homozygous N: heterozygous N:RV phenotypes of the F_2_ population fit a 1:2:1 ratio (χ^2^ = 0.450, *P* = 0.799), suggesting a single dominant gene controlling the restoration of plant vigor.

BSA analysis using 550 SSR and EST-SSR primers from all 17 sunflower LGs among the two parents and the two bulks showed two polymorphic markers, ORS966 and ORS328, on LG 7. Further screening of 30 additional SSR markers on LG 7 identified three polymorphic markers, including two SSR markers, CRT136 and HT520, and one InDel marker ZVG31.

**Figure 2:**
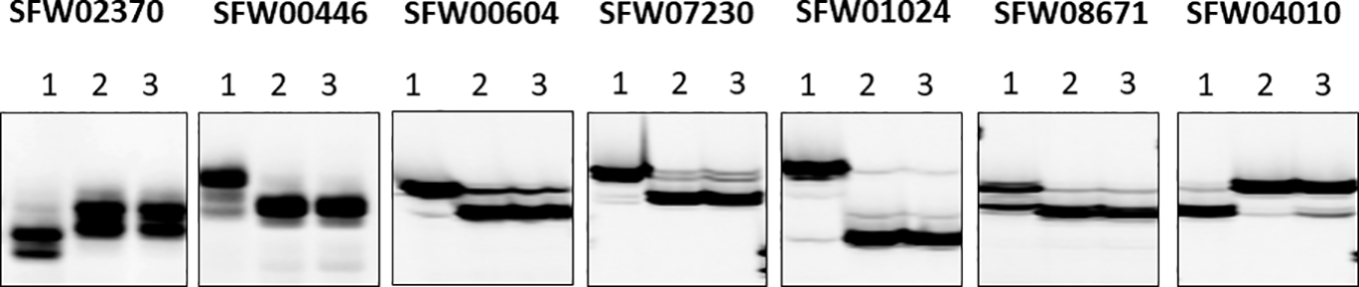
Representative image of seven semi-thermal asymmetric reverse PCR (STARP)-based SNP markers among parents and a bulk of normal F_2_ plants of the cross MOL-RV/HA 821 on a denaturing polyacrylamide gel. (Lane 1) Female parent MOL-RV; (Lane 2) Male parent HA 821; (Lane 3) Bulk-N.

**Figure 3:**
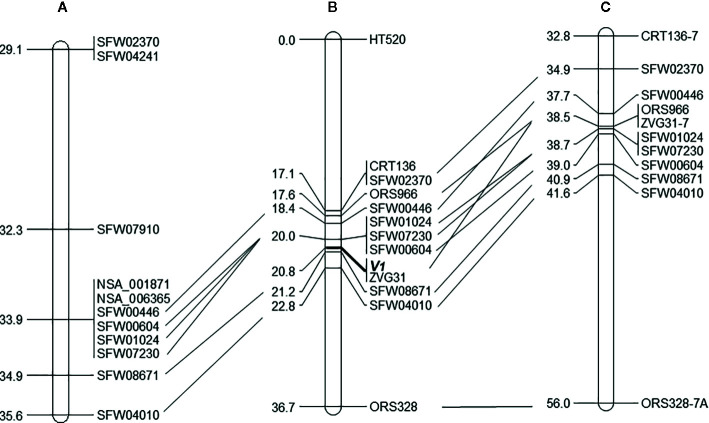
The position of the vigor restoration gene *V_1_* on LG 7 of the sunflower map. **(A)** Scaffold-based genetic map of LG 7 with selected markers (Hulke et al., 2015); **(B)** Mapping result of the *V_1_* gene on LG 7, with 12 linked markers, based on the analysis of 124 F_2_ plants derived from the cross of MOL-RV/HA 821; **(C)** Corresponding region of LG 7 of the genetic map of the sunflower genome based on multiple crosses (Bowers et al., 2012). The same markers are aligned by solid lines among the three maps.

Of the 30 SNP markers tested on the LG 7 map, 18 showed polymorphisms between the two parents and Bulk-N (representative primers shown in **Figure 2**). The polymorphic SNPs were located at the 15.06–42.56 cM region on the scaffold-based genetic map of LG 7 of Hulke et al. (2015). The screening results showed that some markers were far from the *V_1_* gene, with the Bulk-N containing the band from the RV parent. Therefore, the markers from the 26.94–35.55 cM region of the scaffold-based genetic map of LG 7 of Hulke et al. (2015) were used as the focal point to add more SNP markers around the *V_1_* gene. Seven polymorphic PCR-based SNP markers from SFW02370 to SFW04010 were used to genotype the F_2_ mapping population, which were all co-dominant. The sequences and the product length of these STARP markers were shown in **Table 4**. As a result, a linkage map including 12 markers (four SSR, one InDel, and seven SNPs) and the *V_1_* gene was constructed, covering a genetic distance of 36.7 cM (**Figure 3B**). The *V_1_* gene co-segregated with the marker ZVG31, with three SNP markers, SFW01024, SFW07230, and SFW00604, located above *V_1_* on the map at a genetic distance of 0.8 cM, and another SNP marker SFW08671, below it at a distance of 0.4 cM. Comparison of the order of markers on LG 7 containing *V_1_* with those on the LG 7 of Hulke et al. (2015) (**Figure 3A**) and Bowers et al. (2012) (**Figure 3C**) showed the same order for most of the markers, except the order of ORS966, SFW00446, and ZVG31 between **Figures 3B, C**. The order of ORS966 and SFW00446 was reversed between **Figures 3B, C**. The distance between ORS966 and ZVG31 on **Figure 3B** was 3.2 cM, whereas they co-segregated on **Figure 3C**.

**Table 4:** Primers of seven SNP markers on LG 7 mapped in this study.

SNP name	Primer name	Primer sequence (5ʹ→3ʹ)^a^	Product length (bp)^b^
SFW02370	SFW2370F1	[Tail1]GGACGTTTAAGATGACCGAT**T**C***C***	43
	SFW2370F2	[Tail2]GGACGTTTAAGATGACCGATC**T*T***	
	SFW2370R	GGAGGACAGTGTTCGGGTG	
SFW00446	SFW0446F1	[Tail1]GAATTACGCAACGCGAG**T**CA***C***	43
	SFW0446F2	[Tail2]GAATTACGCAACGCGAGC**T**A***T***	
	SFW0446R	ACCATCCGGATTGCATCCTTC	
SFW00604	SFW0604F1	[Tail1]AGTGCAAGCACTAGAATCA**T**C***G***	61
	SFW0604F2	[Tail2]AGTGCAAGCACTAGAATC**C**CC***A***	
	SFW0604R	TGGGCAAGGTTACAACGCTA	
SFW01024	SFW1024F1	[Tail1]GAAACTTAAACAAGTTTTATCGGGT**C**T***C***	82
	SFW1024F2	[Tail2]GAAACTTAAACAAGTTTTATCGGG**C**AT***A***	
	SFW1024R	CGCGAAACGTTTTGATAATGATG	
SFW07230	SFW7230F1	[Tail1]GGGCACGACATAGATGTTC**T*G***	65
	SFW7230F2	[Tail2]GGGCACGACATAGATGTT**T**C***A***	
	SFW7230R	GGCGAAGAGGGAGACACAC	
SFW08671	SFW8671F1	[Tail1]GTGAAGCGAAATTCCATCAA**A**GG***G***	53
	SFW8671F2	[Tail2]GTGAAGCGAAATTCCATCAAG**A**G***A***	
	SFW8671R	TGAGTTGCGTAAATGAGACCGA	
SFW04010	SFW4010F1	[Tail1]GGAAGGCATCATGTTGAG**C**AC***C***	65
	SFW4010F2	[Tail2]GGAAGGCATCATGTTGAGT**C**C***T***	
	SFW4010R	AATTGGCGGTTTTTCCGCTG	

a [Tail1] = GCAACAGGAACCAGCTATGAC-3ʹ; [Tail2] = GACGCAAGTGAGCAGTATGAC-3ʹ. The primers with [Tail1] or [Tail2] are asymmetrically modified allele-specific primers (AMAS-primers). Nucleotide in italics indicates the SNP loci, and the underlined one indicates the modified nucleotide.

b The length was from the design of allele-specific primers for SNPs, which does not include [Tail1] or [Tail2].

### Physical Location of *V_1_* on LG 7

The sequences of the SNP markers and other markers closely linked to *V_1_* were aligned to the reference genome sequences of HA 412-HO and XRQ, respectively (**Table 5**). The seven SFW SNP markers above and below the *V_1_*gene (from SFW02370 to SFW04010) on LG 7 span a 6.7-cM genetic distance on **Figure 3C**, which corresponds to a physical distance of 5.83 and 2.89 Mb on chromosome 7 of the HA 412-HO and XRQ assemblies, respectively. The order of the markers on the genetic maps is generally consistent with their physical order on the genome assemblies of HA 412-HO and XRQ, respectively, except for the order of the three co-segregated SNP markers SFW01024/SFW07230/SFW00604, and that of ZVG31 and SFW08671. SFW01024 was located above SFW00604 and SFW07230 on the HA 412-HO assembly, whereas it was below them on the XRQ assembly. The order of ZVG31 and SFW08671 was reversed compared between the HA 412-HO and XRQ assemblies. The *V_1_*gene was mapped between SNP markers SFW01024/SFW07230/SFW00604 and SFW08671 on LG 7, spanning a 1.2-cM genetic distance on **Figure 3B**. The physical distance between the two closest flanking SNP markers corresponds to 0.56 Mb at the 91,735,216–92,294,131 bp region (between SNP markers SFW07230 and SFW08671) on the HA 412-HO assembly, and 1.37 Mb at the 86,351,353–87,722,487 bp region (between SNP markers SFW01024 and SFW08671) on the XRQ assembly, respectively (**Table 5**). Interestingly, preliminary sequence analysis at the target region on the XRQ sunflower genome showed a chloroplastic NifU-like nitrogen fixation protein 3 gene, located at the 87,181,582–87,185,092 bp on LG 7, with a length of 3,511 bp. In addition, two other cytoplasmic-related genes were also detected at its nearby region of 87,185,542–87,205,817 bp. Previously, Yabe et al. (2004) reported a mutant *Arabidopsis* lacking a chloroplast-localized NifU-like nitrogen fixation protein AtCnfU-V exhibited a dwarf phenotype with faint pale-green leaves and had drastically impaired photosystem I accumulation.

**Table 5:** Genetic and physical positions of the SNP and other markers linked to the *V_1_* gene on linkage group (LG) 7.

Marker	Genetic position on *V_1_* map (cM)	Genetic position on Hulke et al. (2015) map (cM)	Genetic position on Bowers et al. (2012) map (cM)	HA 412-HO Assembly		XRQ Assembly	
				Start	End	Start	End
SFW02370	17.1	29.1	34.9	87,302,908	87,303,027	84,925,208	84,925,327
ORS966	17.6		38.5	88,832,583	88,832,951	85,352,367	85,352,735
SFW00446	18.4	33.9	37.7	91,176,478	91,176,596	85,936,946	85,937,064
SFW00604	20.0	33.9	39.0	91,734,940	91,734,821	85,849,228	85,849,109
SFW07230	20.0	33.9	38.7	91,735,216	91,735,100	85,849,507	85,849,388
SFW01024	20.0	33.9	38.7	91,493,739	91,493,621	86,351,353	86,351,228
*V_1_*	20.8						
ZVG31	20.8		38.5	92,650,577	92,650,929	86,535,616	86,535,968
SFW08671	21.2	34.9	40.9	92,294,250	92,294,131	87,722,606	87,722,487
SFW04010	22.8	35.6	41.6	93,133,863	93,133,745	87,811,477	87,811,595

The length of LG 7 in the sunflower physical map is 109,221,022 bp for HA 412-HO and 103,871,911 bp for XRQ.

### Vigor Restoration of Cultivated Sunflower for Progenies With *H. giganteus* Cytoplasm

In the process of transferring Sclerotinia resistance and other useful genes from wild perennial *Helianthus* species into cultivated sunflower, we also observed the RV plants in the progenies derived from the crosses involving the perennial *H. giganteus*. Segregation of N and RV plants was observed starting with the BC_3_F_1_ generation of *H. giganteus*/HA 89 when the chromosome numbers ranged from 34 to 41. Approximately 50% of the BC_4_F_1_ plants had 2n = 34, while the remainder ranged from 2n = 35 to 36. Since HA 89 does not contain a vigor restoration gene for the vigor-reducing perennial species cytoplasm, normal BC_4_F_1_ plants with 2n = 34 must have obtained the vigor restoration gene from *H. giganteus*, and the BC_5_F_1_ progeny segregation of 33 N to 26 RV plants fit the 1 N to 1 RV ratio (χ^2^ = 0.831, *P* = 0.362), indicating a single dominant gene control of vigor restoration.

The amphiploid NMS HA 89/*H. maximiliani* 1631 provided the male-fertility restoration gene *Rf_4_* to CMS GIG2 (Feng and Jan, 2008). Therefore, the F_2_ population derived from a normal-vigor and fertile F_1_ plant of cross CMS GIG2//CMS GIG2/(NMS HA 89/*H. maximiliani* 1631, Amp) segregated for both vigor and male-sterility. This F_2_ population included 82 N and 35 RV plants, which was consistent with the 3 N to 1 RV segregation ratio expected with single gene control of vigor restoration (χ^2^ = 1.507, *P* = 0.220). Meanwhile, the 79 male-fertile to 31 male-sterile progenies also fit a 3 MF to 1 MS ratio (χ^2^ = 0.594, *P* = 0.441), indicating a single gene control of fertility restoration. When the two traits, vigor and fertility restoration, were combined, the resulting 53, 26, 22, and nine plants of normal-vigor male-fertile, normal-vigor male-sterile, reduced vigor male-fertile, and reduced vigor male-sterile plants, respectively, fit a 9:3:3:1 ratio (χ^2^ = 0.748, *P* = 0.331), indicating the *V* and the *Rf* genes are not linked.

Progeny segregation of 14 normal plants of CMS GIG2/HA 821 crossed with HA 89 is shown in **Table 6**. Ten populations with segregation ratios of N to RV vigor plants fit the 3 N to 1 RV ratio, and four fit the 1 N to 1 RV ratio. CMS GIG2 was a normal plant, but its vigor restoration gene was heterozygous. The segregation of N and RV plants in all these progenies indicated that the vigor restoration gene derived from *H. giganteus* 1934 is different from the *V_1_* gene commonly existing in cultivated lines, designated *V_2_* here.

**Table 6:** Segregation of normal (N) and reduced-vigor (RV) plants in the progeny of cross CMS GIG2/HA 821//HA 89.

Pedigree	No. plants		Theoretical segregation ratio tested	χ^2^	*P*-value
	N	RV	N:RV		
CMS GIG2/HA 821//HA 89	22	2	3:1	3.556	0.059
CMS GIG2/HA 821//HA 89	17	7	3:1	0.222	0.637
CMS GIG2/HA 821//HA 89	21	4	3:1	1.080	0.299
CMS GIG2/HA 821//HA 89	10	15	1:1	1.000	0.317
CMS GIG2/HA 821//HA 89	13	11	1:1	0.167	0.683
CMS GIG2/HA 821//HA 89	17	7	3:1	0.222	0.637
CMS GIG2/HA 821//HA 89	15	9	3:1	2.000	0.157
CMS GIG2/HA 821//HA 89	13	11	1:1	0.167	0.683
CMS GIG2/HA 821//HA 89	18	7	3:1	0.120	0.729
CMS GIG2/HA 821//HA 89	16	5	3:1	0.016	0.900
CMS GIG2/HA 821//HA 89	19	5	3:1	0.222	0.637
CMS GIG2/HA 821//HA 89	15	8	3:1	1.174	0.279
CMS GIG2/HA 821//HA 89	12	13	1:1	0.040	0.841
CMS GIG2/HA 821//HA 89	17	7	3:1	0.222	0.637
Homogeneity			3:1	8.640	0.471
Homogeneity			1:1	1.333	0.721

### Other *V* Genes Derived From Wild Perennial *Helianthus* Species

The *V* genes derived from *H. hirsutus* and *H. giganteus* were compared to all other detected *V* genes. The F_1_ progenies of vigor-reducing lines with cytoplasms of *H. grosseserratus* (GRO-RV), *H. angustifolius* (ANG-RV), *H. salicifolius* (SAL-RV), *H. hirsutus* (HIR-RV), and *H. pauciflorus* (PAU-RV) substituted with the nuclear genomes of HA 89 or HA 410, pollinated by six normal lines having homozygous *V* genes (HA 821, RF GIG2-MAX 1631, HIR, ANN PI 649856, ANN Bulk and PET Bulk) were all normal (**Table 7**). The results of all normal progeny clearly indicated the common cytonuclear interaction defects in the progeny plant with perennial *Helianthus* cytoplasms and annual nuclear genomes, and the common function of vigor restoration genes from different sources of HA 821, *H. giganteus*, *H. hirsutus*, two *H. annuus*, and *H. petiolaris*.

**Table 7:** Segregation of normal (N) and reduced-vigor (RV) progenies of F_1_s derived from RV lines of HA 89 or HA 410 with five perennial *Helianthus* vigor-reducing cytoplasms crossed with six homozygous vigor restoration lines.

Parents	HA 821	RF GIG2-MAX 1631	HIR	ANN PI 649856	ANN Bulk	PET Bulk
GRO-RV	24:0	26:0	19:0	22:0	21:0	22:0
ANG-RV	23:0	20:0	23:0	24:0	17:0	18:0
SAL-RV	21:0	24:0	23:0	13:0	16:0	9:0
HIR-RV	20:0	20:0	23:0	20:0	14:0	14:0
PAU-RV	25:0	24:0	23:0	24:0	24:0	24:0

The segregation of N and RV progenies of testcross progenies derived from the partial half-diallel crosses among the six homozygous or heterozygous vigor restoration lines are shown in **Table 8**. No segregation of plant vigor was observed in the progenies derived from the crosses involving four sources with normal vigor (HA 821, ANN Bulk, ANN PI 649856, and PET Bulk), indicating that they all contained the same *V_1_*gene for plant vigor restoration. Meanwhile, no segregation of plant vigor was observed in the progenies derived from the cross HIR/RF GIG2-MAX 1631 and the segregation of plant vigor was observed in the progenies derived from the crosses between HIR or RF GIG2-MAX 1631 and the four *V_1_* sources, suggesting *H. hirsutus* PI 547174 and *H. giganteus* 1934 had the same *V_2_* gene (**Table 8**).

**Table 8:** Segregation of normal (N) and reduced-vigor (RV) progenies of testcross F_1_s derived from the partial half-diallel cross of females with homozygous or heterozygous *V* genes derived from six sources (HA 821, *H. giganteus*, *H. hirsutus*, *H. annuus* bulk, *H. annuus* PI 649856, and *H. petiolaris* bulk), pollinated onto CMS RIGX-RV (*v_1_v_1_v_2_v_2_*).

Parents	HA 821 (*V_1_V_1_*)	RF GIG2-MAX 1631 (*V_2_V_2_*)	ANN Bulk (*V_1_V_1_*)	ANN PI 649856 (*V_1_V_1_*)
	N: RV			
RF GIG2-MAX 1631^a^ (*V_2_V_2_*)	92:32			
HIR^a^ (*V_2_V_2_*)	77:23	101:0		
GRO-RV/ANN Bulk^b^ (*V_1_v_1_*)	198:0	106:42		
GRO-RV/ANN PI 649856^b^ (*V_1_v_1_*)	143:0	168:75	128:0	
GRO-RV/PET Bulk^b^ (*V_1_v_1_*)	93:0	145:42	170:0	137:0

a The female parents with homozygous V genes were used for F_1_ production, and one testcross family was used for segregation analysis.

b The female parents with heterozygous V genes from wild H. annuus (i.e. ANN Bulk and ANN PI 649856) and H. petiolaris (i.e. PET Bulk) were used to cross with four homozygous V gene sources, with 4–5 testcross families used for segregation analysis. The numbers for N or RV progenies were combined according to 3:1 segregation ratio or no segregation. The families with 1:1 segregation ratio were not shown in this table.

## Discussion

### The Existence of Vigor Restoration (*V*) Genes in Both Wild *Helianthus* Species and Cultivated Sunflower

Cytonuclear interactions could act as a source of variation for interspecific hybridization and may drive speciation (Levin, 2003). A study on wheat alloplasmic lines carrying the cytoplasm of *Aegilops mutica* showed that novel nuclear-cytoplasmic interactions can potentially trigger an epigenetic modification cascade in nuclear genes, which eventually change physiological traits, such as dry matter weight (Soltani et al., 2016). The research on *Arabidopsis* cytolines (each combining the nuclear genome of a natural variant with the cytoplasmic genomes of a different variant) indicated that genetic variation in organelle genomes could impact three seed physiological traits including dormancy, germination performance, and longevity (Boussardon et al., 2019). Their results also showed that natural parental accessions had contrasted contributions to the cytonuclear effect on germination phenotype depending whether they provided the nuclear or cytoplasmic genomes. In this study, we also detected a reduction of plant vigor with pale-green leaves and stunted growth in interspecific progenies involving different perennial *Helianthus* species, but only when using the wild perennial species as the maternal parent and cultivated sunflower as the paternal parent. No reduction of plant vigor was observed in the reciprocal crosses, or in progenies derived from the crosses involving wild annual *Helianthus* species. Therefore, a common cytoplasmic-nuclear interaction defect commonly exists in alloplasmic lines derived from wild perennial *Helianthus* species.

Since the vigor restoration gene was thought to exist in only the perennial *Helianthus* species, the high frequency of *V* in cultivated lines was not expected. Because the cytoplasms of other annual species do not cause adverse interaction with nuclear genes in cultivated sunflower, the CMS PET1 cytoplasm (derived from *H. petiolaris*, an annual species) (Leclercq, 1969) has been used successfully for hybrid sunflower production for about 50 years. However, if there is ever a need to use perennial *Helianthus* species cytoplasms, the abundance of *V* genes in cultivated germplasm lines should not hinder the utilization of perennial species cytoplasms in sunflower breeding programs. Our earlier work only demonstrated that wild annual *Helianthus* species did not produce RV plants, likely because they didn’t have vigor-reducing cytoplasms. For the tested materials in this study, the reduced-vigor trait was only observed in the progenies for the crosses using wild perennial species as the maternal parent, not for the two annual species. The current study has shown that the *V* genes in wild annual *Helianthus* species, including *H. annuus* and *H. petiolaris*, are the same as the *V_1_* gene that commonly exists in the cultivated sunflower lines. Future research with diverse sources of annual and perennial species may be necessary to determine whether there is deficiency in their cytoplasms causing reduced vigor and the evolutionary role of *V* gene for the annual *Helianthus* species.

Similarly, an explanation for the high frequency of a *V* gene in cultivated lines without any obvious selective advantage is not clear. Since *H. tuberosus* has been used extensively in the improvement of cultivated sunflower (Fick and Miller, 1997), it is also possible that the *V* gene is tightly linked with genes controlling desirable agronomic traits and was simultaneously selected and maintained in those lines. As more sunflower genes are mapped, the prevalence of the *V* gene in cultivated lines may eventually be more clearly explained.

### *H. hirsutus* PI 547174 and *H. giganteus* 1934 Had a Different *V* Gene Than Other *Helianthus* Species

The crosses involving five perennial *Helianthus* vigor-reducing cytoplasms with six homozygous vigor restoration lines have assessed the commonality of the cytoplasmic-nuclear interaction defect of RV cytoplasms from different perennial *Helianthus* species, as well as the vigor restoration genes. The segregation patterns of the progenies of the partial half-diallel crosses among the six homozygous/heterozygous vigor restoration lines indicated that *H. hirsutus* PI 547174 and *H. giganteus* 1934 had a different *V* gene than other *Helianthus* species. For the convenience of future research, we have designated the vigor restoration gene identified in 1992 (Jan, 1992) and those identified in cultivated lines as *V_1_*, and the *V* from *H. giganteus* and *H. hirsutus* as *V*_2_, respectively. Although the two *V* genes are located on different loci, they both can restore the plant vigor of the progeny containing different cytoplasms, which suggests that they can compensate for a common cytonuclear interaction defect causing reduced plant vigor.

The segregation of normal plants in the F_1_ and F_2_ progeny of MOL-RV/P21 indicated that P21 has the *V* gene to restore the reduced vigor trait (**Tables 2** and **3**). P21 has been used to produce several amphiploids for sunflower improvement (Liu et al., 2013; North Dakota State University Foundation Seedstocks (NDSUFS), website: https://www.ag.ndsu.edu/fss/ndsu-varieties/usda-sunflower-inbred-lines). Therefore, in the study of *V* genes in sunflower, one needs to avoid adding more *V* genes by carefully checking their pedigree not involving P21 in amphiploids. The *V* gene discovered in other sources will need to be compared with the *V*_1_ and *V*_2_ genes for allelism, as well as their effectiveness for restoring other perennial RV cytoplasms. Further study of the RV and its restoration caused by the cytoponuclear gene interaction in multi-species may help to elucidate the speciation of annual and perennial *Helianthus* species.

### The *V_1_* Gene Was Mapped on LG 7 Using SNP and Other Markers

According to BSA screening results between Bulk-N and Bulk-RV using the already mapped SSR/InDel markers, the *V_1_* gene was mapped to LG 7. However, only five markers were linked to *V_1_*. With the aim of adding more markers close to *V_1_*, according to the linkage maps with high-density of SNPs, 30 SNP markers were selected to design PCR-based SNP markers. The markers in a focused region from 26.94 to 35.55 cM on the scaffold-based genetic map of LG 7 of Hulke et al. (2015) were used for further genotyping of the F_2_ population. Seven co-dominant SNP markers were successfully added to the map on both sides of *V_1_*. Using the flanking sequences of the SNP markers and primer sequences of other markers, the *V_1_* gene has been located onto the physical map of chromosome 7 of the sunflower genome, i.e., a 0.56 Mb region on the HA 412-HO assembly, and a 1.37 Mb region on the XRQ assembly. The tightly linked molecular markers identified in this study will facilitate the marker-assisted selection for the lines with vigor restoration genes at the early stages of sunflower breeding, especially utilizing the CWR.

The pattern of cytonuclear interactions is the result of a long-term coevolution between nuclear and organellar genomes under selection pressure, which is essential for the proper function of plant cells (Postel and Touzet, 2020). When using organellar markers to evaluate phylogenetic relationships for characterizing genetic diversity, mitochondrial and chloroplast genes often show markedly different phylogenetic patterns from nuclear markers, which is called “cytonuclear discordance” (Lee-Yaw et al., 2019). Phylogenetic analyses using whole-chloroplast sequence data in combination with over 1000 nuclear SNPs in wild annual *Helianthus* indicate that cytonuclear discordance is widespread both among species and among individuals within species. Since mitochondria and chloroplasts affect key physiological processes, selection may have played a role in driving patterns of plastid variation (Lee-Yaw et al., 2019). In this study, the existence of *V* genes to different *Helianthus* cytoplasms and the vigor restoration ability across the *Helianthus* species provides another piece of evidence for cytonuclear discordance in *Helianthus* genus. Since there is clear distinction between the nuclear and chloroplast of annual and perennial *Helianthus* species (Stephens et al., 2015; Lee-Yaw et al., 2019), the *V* genes contained in the nuclear genomes of both annual and perennial species suggest that the *V* genes may have evolved before the speciation of annual and perennial *Helianthus* species.

Many cytonuclear incompatibilities are caused by plastid-nuclear incompatibilities, which has been reported in many flowering plants, such as in *Passiflora*, *Oenothera* and *Pisum*. Such incompatibilities often produce lutescent, chlorosis/virescence, or variegation, because of a decreased photosynthetic function of plastid complex malfunction in the plants (Greiner et al., 2011; Postel and Touzet, 2020). On the other hand, the incompatibility between mitochondrial and the nuclear genomes will often cause CMS (Postel and Touzet, 2020). As a result, several corresponding nuclear *Rf* gene have been identified and molecularly mapped in sunflower for different CMS sources, such as *Rf_1_* for CMS PET-1, *Rf_4_* for CMS GIG2, and *Rf_6_* for CMS 514A (Horn et al., 2003; Feng and Jan, 2008; Liu et al., 2013).

In this study, we have mapped the vigor restoration gene *V_1_* to the LG 7 of HA 412-HO and XRQ sunflower assemblies. With these targeted regions containing the *V_1_* gene, identification of more molecular markers, such as SNPs or SSR, according to the genomic DNA sequences of the sunflower genome will facilitate fine mapping of the *V_1_* gene, as well as future map-based cloning of the *V_1_* gene. Further fine-mapping, detailed analysis of the genes contained in the corresponding regions of the two assemblies, plus using microarray and RNA-Seq (RNA sequencing) techniques (Wang et al., 2009) will help to identify candidate genes for plant vigor restoration. Using other methods such as RT-PCR, gene knock-out, or gene-editing will confirm the function of the gene, and thus will reveal the mechanism for vigor reduction and restoration in sunflower and will help to understand the interaction between the cytoplasm and nuclear genes. Therefore, the inheritance study and molecular mapping of vigor restoration gene in this study will also provide evidence for the speciation of annual and perennial *Helianthus* species. Finally, the results of these studies will provide the basis for better and more efficient utilization of sunflower CWR in crop improvement.

## Data Availability Statement

The raw data supporting the conclusions of this article will be made available by the authors, without undue reservation, to any qualified researcher.

## Author Contributions

C-CJ and ZL conceived and designed the research. ZL, GU, C-CJ performed the experiments, ZL and C-CJ analyzed the data and ZL, C-CJ and GS wrote the manuscript. All authors contributed to the article and approved the submitted version.

## Funding

The project was supported by the USDA-ARS National Sclerotinia Initiative, Grant No. 3060-21220-028-00D, the USDA-ARS CRIS Project No. 3060-21000-043-00D, and the Heilongjiang Postdoctoral Fund of China, Grant No. LBH-Z14190. Mention of trade names or commercial products in this article is solely for the purpose of providing specific information and does not imply recommendation or endorsement by the U.S. Department of Agriculture. USDA is an equal opportunity lender, provider, and employer.

## Conflict of Interest

The authors declare that the research was conducted in the absence of any commercial or financial relationships that could be construed as a potential conflict of interest.
